# Are Preexisting Retinal and Central Nervous System-Related Comorbidities Risk Factors for Complications Following Robotic-Assisted Laparoscopic Prostatectomy?

**DOI:** 10.1590/S1677-5538.IBJU.2014.0464

**Published:** 2015

**Authors:** David Chalmers, Antonio Cusano, Peter Haddock, Ilene Staff, Joseph Wagner

**Affiliations:** 1 Department of Urology, University of Connecticut, Farmington, USA and Research Group, Hartford Hospital, Hartford, USA; USA and Research Group, Hartford Hospital, Hartford, USA; 2 Urology Division, Hartford Healthcare Medical group, Hartford, USA

**Keywords:** Laparoscopy, Robotic Surgical Procedures, complications [Subheading], Comorbidity

## Abstract

**Purpose:**

To assess whether retinal and central nervous system (CNS) comorbidities are risk factors for complications following robotic assisted laparoscopic prostatectomy (RALP).

**Materials and Methods:**

A retrospective review of our RALP database identified 1868 patients who underwent RALP by a single surgeon between December 10, 2003-March 14, 2014. We hypothesized that patients with preexisting retinal or CNS comorbidities were at a greater risk of suffering retinal and CNS complications following RALP. Perioperative complications and risk of recurrence were graded using the Clavien and D'Amico systems, respectively.

**Results:**

40 (2.1%) patients had retinal or CNS-related comorbidities, of which 15 had a history of retinal surgery and 24 had a history of cerebrovascular accident, aneurysm and/or neurosurgery. One additional patient had a history of both retinal and CNS events.

Patients with retinal or CNS comorbidities were significantly older, had elevated PSA levels and CCI (Charlson Comorbidity Index) scores than the control group. Blood loss, length of stay, surgical duration, BMI, diagnostic Gleason score and T-stage were not statistically different between groups.

No retinal or CNS complications occurred in either group. The distribution of patients between D'Amico risk categories was not statistically different between the groups. There was also no difference in the incidence of total complications between the groups.

**Conclusions:**

RALP-associated retinal and CNS complications are rare. While our RALP database is large, the cohort of patients with retinal or CNS-related comorbidities was relatively small. Our dataset suggests retinal and CNS pathology presents no greater risk of suffering from perioperative complications following RALP.

## INTRODUCTION

Since the first procedure was performed in 2000, robotic-assisted prostatectomy has become more commonplace and is currently utilized in >80% of prostate cancer surgeries (1, 2). Robotic systems have been introduced into an array of surgical settings with the advantages of three-dimensional vision, ten-fold magnification, Endowrist technology and tremor filtration. Clinical benefits include shorter hospital stay, reduced blood loss and a reduced frequency of blood transfusions (3). RALP surgery has evolved rapidly, with modifications and refinements in technical and procedural steps over the last decade. Since RALP is now the dominant modality for surgically treating localized prostate cancer (PCa) in the United States (3), it is of clinical interest to understand the perioperative risks in patients with preexisting comorbidities elected to undergo this procedure.

An inherent element of the preparation for RALP surgery is placing the patient in a steep (30-45°), supine, head-down Trendelenburg position. While this effectively allows gravity to draw the abdominal viscera away from the operative field (4), prolonged periods in this position (combined with pneumoperitoneum) can have significant hemodynamic, respiratory and cerebrovascular side effects (e.g. venous stasis, facial and laryngeal edema, venous gas embolisms and posterior ischemic optic neuropathy) (5). These can be exacerbated in the setting of abdominal insufflation, acute blood loss, and prolonged operating times (5).

Posterior ischemic optic neuropathy following robotic-assisted surgery has been previously reported (6). An elevation in intraocular pressure (IOP) during RALP has also been documented (7, 8). Elevations in IOP are known to be exacerbated by surgical blood loss (7, 8). Similarly, elevated intracranial pressure (ICP) resulting from an increase in venous pressure (that decrease cerebral venous draining and increase cerebral blood and cerebral spinal fluid volume) has been related to sustained periods of Trendelenburg positioning (9). Changes in IOP and ICP may have a significant clinical impact in surgical patients with retinal and CNS comorbidities, including those with a history of retinal detachment and surgery, cerebrovascular accident (CVA), cerebral aneurysm or neurosurgery.

While retinal and CNS-related complications appear to be relatively rare, such perioperative events can have significant morbidity and impact quality of life. There are limited published data that directly address whether RALP–Trendelenburg positioning may contribute as an etiologic factor in eliciting these potentially significant complications. In the present study, we compared the incidence of overall complications between a control group of patients who underwent RALP with steep Trendelenburg positioning and a patient cohort with preexisting retinal or CNS-related comorbidities undergoing an identical RALP procedure during a 10 year contemporary time period.

## MATERIALS AND METHODS

### Institutional review board approval

The study design and protocol was reviewed and approved by the Hartford Hospital Institutional Review Board (IRB).

### Study design

We undertook a retrospective review of our IRB-approved, prospectively-maintained database to identify patients who underwent RALP surgery by a single surgeon during a 10 year contemporary time period (December 10, 2003–March 14, 2014). Patients with a history of preexisting retinal or CNS-related comorbidities or events (who were hypothesized to be at greater risk of suffering a retinal or CNS-related event following RALP) were selected and formed the study cohort. Patients with glaucoma were not included in this study as this has been examined previously (10). For comparative purposes, the remaining patients without a history of vascular-related retinal or CNS-related comorbidities formed a control group. All patients with pre-existing CNS or ocular pathology are warned of the hypothetical risks of undergoing RALP and are provided with all available evidence based material to make an informed decision. However, patients were not actively excluded from this study because of these comorbidities.

### Surgical procedure

In preparation for RALP surgery, patients were positioned in a modified dorsal lithotomy, in steep Trendeleburg (consistently at-45 degrees from horizontal for all patients) with arms tucked to the side. Body areas considered at elevated risk for sustaining compression-related injury during surgery received additional padding, while shoulder bolsters or more recently a Hugavac device (Allen Medical, USA) were used to secure the patients position.

### Perioperative indices

Patients were observed and interviewed during the immediate postoperative period to determine and record any symptomatology consistent with neuropathy, paresthesias, and paralysis. Any complications were graded according the Clavien grading system (11).

The D'Amico risk of recurrence following treatment was assessed using clinical TNM stage, biopsy Gleason score, and preoperative prostate–specific antigen (PSA) levels (12). D'Amico scores were used to stratify patients into those with low, intermediate, or high-risk of recurrence after surgery. Patient age, body mass index (BMI), operative time, estimated blood loss (EBL), D'Amico risk and pathologic stage were compared between the control group and patients in the study group who experienced retinal and/or CNS-related symptoms.

The incidence of complications and comorbidities, and categorical descriptions of patient characteristics were analyzed with chi-square tests of proportion. Continuous variables (e.g. age, BMI, robot and operative time) were analysed with t-tests for independent groups. Ordinal measures (such as CCI scores) and continuous measures that did not meet assumptions of normality were analysed with Wilcoxon Ranked Sum tests.

All statistical analyses were performed using SPSS v21.0 (SPSS, Inc., Chicago, IL, USA.).

## RESULTS

### Patient demographics

A total of 1868 patients who underwent RALP with steep Trendelenberg positioning were included in the study. Of these, 40 (2.1%) had a history of a prior retinal or CNS-related event or comorbidity, of which 15 (37.5%) had a history of prior retinal surgery and 24 (60%) had a history of cerebrovascular accident, aneurysm and/or neurosurgery. One additional patient (2.5%) had a history of both retinal and CNS events. The remaining 1828 (97.9%) patients had no prior history of suffering either a retinal or CNS-related event.

Patients with preexisting retinal and CNS comorbidities were significantly older than the control group (62.1±7.1 vs. 59.7±6.6 years, respectively; P=0.024). CCI scores in patients with CNS or retinal comorbidities were significantly higher than the control group (3 vs. 4; p<0.001; Table-1). In contrast, BMI was not significantly different between the two groups (28.2±4.6 vs. 28.2±3.6 kg/ m^2^; p=0.992). There was no significant difference in either the total surgical time or robotic surgery time between the two groups (p=0.963 and 0.827; respectively; Table-1). In addition, there was no significant difference in estimated blood loss or length of hospital stay between the study and control groups (p=0.313 and 0.362, respectively; Table-1). The distribution of patients between low, intermediate and high categories of D'Amico risk was not statistically different between the control and study cohort of patients (p=0.564; Table-2).

**Table 1:** Patient demographics and clinical data of patients who underwent RALP.

			All patients	Control Group	Study Group	p (control vs. study group)
Number of patients (n)			1868	1828	40	-
Age (mean) (years)			59.7±6.7	59.7±6.6	62.1±7.1	0.024
BMI (mean) (kg/m2)			28.2±4.5	28.2±4.6	28.2±3.6	0.992
Total operative time (min) (mean, median, IQR) (min)			180.9±44.9	180.9±44.9	181.3±46.2	0.963
Robotic surgical time (min) (mean, median, range) (min)			142.3±40.2	142.3±40.2	143.7±43.2	0.827
Initial PSA (Median, IQR) (ng/mL)			5.3±4.2 (3.5-6.5)	4.8 (3.5-6.5)	4.3 (3.0-5.6)	0.039
D'Amico Risk category (n; %)		Low	835 (44.7)	818 (44.8)	17 (42.5)	0.564
		Intermediate	806 (43.1)	790 (43.3)	16 (40.0)	
		High	225 (12)	218 (11.9)	7 (17.5)	
		Unknown	2 (0.1)	2 (0.1)	0 (0)	
CCI score (median; IQR)			3 (2-3)	3 (2-3)	4 (3-4)	<0.001
Estimated surgical blood loss (median, IQR) (mL)			200 (125-375)	200 (130-393.8)	200 (100-337.5)	0.313
Length of stay (median, IQR) (days)			1 (1-2)	1 (1-2)	1 (1-2)	0.362
Gleason score (n; %)	Diagnostic biopsy	≤6	912 (49.1)	896 (49.1)	16 (48.5)	0.391
		7	770 (41.4)	754 (41.3)	16 (48.5)	
		≥8	176 (1)	175 (9.6)	1 (3)	
	Pathology biopsy	≤6	483 (25.9)	473 (26)	10 (25)	0.797
		7	1235 (66.4)	1207 (66.4)	28 (70)	
		≥8	141 (7.6)	139 (7.6)	2(5)	
Tumor stage (median, range)				5 (5-5)	5 (5-5)	0.213

**Table 2:** Retinal and CNS-related comorbidities of patients who underwent RALP.

Preexisting comorbidity	Number of patients with comorbidity (% total)			p (control vs. study group)
	All patients	Control Group	Study Group	
CNS	24 (1.3)	-	24	-
Retinal	15 (0.8)	-	15	-
CNS+retinal	1 (0.1)	-	1	-
CAD	0 (0)	0 (0)	0 (0)	-
CHF	1 (0.1)	0 (0)	1 (2.5)	0.021
COPD/Pulmonary	128 (6.9)	124 (6.8)	4 (10.0)	0.349
Diabetes	149 (8)	146 (8.0)	3 (7.5)	1.0
Connective Tissue	1 (0.1)	1 (0.1)	0 (0)	1.0
Peptic Ulcer Disease	119 (6.4)	116 (6.3)	3 (7.5)	0.739
Renal Insufficiency	51 (2.7)	48 (2.6)	3 (7.5)	0.093
Cerebrovascular	26 (1.4)	2 (0.1)	24 (60.0)	<0.001
Peripheral Vascular	100 (5.4)	96 (5.3)	4 (10.0)	0.163
Lymphoma	0 (0)	0 (0)	0 (0)	-
MI	90 (4.8)	90 (4.9)	0 (0)	0.259
Liver	1 (0.1)	1 (0.1)	0 (0)	1.0
Any	544 (29.1)	516 (28.2)	28 (70.0)	<0.001

No retinal or CNS-related perioperative complications were reported in either the study or control groups. There was no significant difference in the incidence of patients in the control and study groups suffering any perioperative complication (p=0.50; Table-3). The rates of other individual perioperative complications between the two groups were also not statistically different (Table-3). There was no statistical difference in the incidence of minor (Clavien 1-2) or major (Clavien 3-5) complications between the control and study group (Table-4).

**Table 3:** Incidence of complications in patients following RALP surgery.

Complication	Number of patients with complication (% total)			p (control vs. study group)
	All patients	Control Group	Study Group	
CNS	0 (0)	0 (0)	0 (0)	-
Retinal	0 (0)	0 (0)	0 (0)	-
Cardiac	3 (0.2)	3 (0.2)	0 (0)	1.0
Respiratory	5 (0.3)	5 (0.3)	0 (0)	1.0
Genito-urinary	121 (6.5)	118 (6.5)	3 (7.5)	0.741
Gastrointestinal	48 (2.6)	47 (2.6)	1 (2.5)	1.0
Infection	44 (2.4)	43 (2.4)	1 (2.5)	0.619
Vascular	44 (2.4)	42 (2.3)	2 (5)	0.242
Nausea	0 (0)	0 (0)	0 (0)	-
Misc. Medical	13 (0.7)	12 (0.7)	1 (2.5)	0.246
Misc. Surgical	46 (2.5)	46 (2.5)	0 (0)	0.623
Other	0 (0)	0 (0)	0 (0)	-
Death	0 (0)	0 (0)	0 (0)	-
ANY	268 (14.3)	261 (14.3)	7 (17.5)	0.50

**Table 4:** Clavien grading of complications in control and study groups.

Clavien grading	Number of patients (%)		P (control vs. study group)
	Control group	Study group	
1	70 (3.8)	0 (0)	0.4
2	92 (5)	4 (10)	0.146
3a	32 (1.8)	0 (0)	1.0
3b	108 (5.9)	3 (7.5)	0.729
4	12 (0.7)	1 (2.5)	0.246
5	0 (0)	0 (0)	-
Minor complications (Clavien 1-2)	156 (8.5)	4 (10.0)	0.772
Major Complications (Clavien 3-5)	146 (8.0)	4 (10)	0.557

## DISCUSSION

In comparison to open surgery, robotic-assisted laparoscopic prostatectomy is associated with beneficial improvements in intraoperative blood loss, postoperative recovery time, hospitalization period and postoperative pain (1–3). However, laparoscopic prostate surgery requires steep Trendelenburg positioning of the patient to create a clear, accessible surgical window. Prolonged periods in this position have the potential to contribute to perioperative retinal and CNS-related complications by evoking changes in IOP, ICP, edema and stretching or compression of nervous tissue (4–8). As such, it is reasonable to hypothesize that patients with preexisting retinal or CNS–related injuries or comorbidities may be at an elevated risk of suffering additional retinal or CNS complications in the immediate perioperative period following RALP.

Supine, head-down Trendelenberg positioning allows gravity to draw the abdominal viscera away from the operative field, retracting the bowels and improving surgical access. The cardiovascular and neurophysiological impact of Trendelenberg positioning (e.g. increase in intraocular pressure, extremity nerve injury, peripheral pain) during robotic surgery is of clinical relevance, particularly in elderly patients and those with preexisting cardiovascular and CNS-related comorbidities.

Data from our study illustrate that patients with preexisting retinal and CNS comorbidities undergoing RALP surgery were at no greater risk of suffering retinal and CNS-related complications compared to a control group of patients. There were no instances of retinal or CNS-related complications in either the 1833 control group patients or the 40 patients with a history of a prior retinal or CNS-related comorbidity. Despite the large size of our RALP patient database, the study group was relatively small due to the low incidence of preexisting retinal and CNS-related comorbidities in our prostatectomy patient population. Furthermore, the absence of retinal or CNS-related perioperative events in 1828 control group patients suggests that the rate of these types of complications following RALP is inherently low. As such, it may not be surprising that the rate of retinal and CNS–related complications was not elevated in our ‘at risk’ study group.

Perioperative increases in intraocular pressure are of particular concern in individuals with chronically elevated IOP, or in high-risk populations that have a diminished tolerance for deviations from normal physiology. These elevated risk factors in certain patient populations may contribute to ischemic optic neuropathy following RALP (6). Evidence from spinal surgery literature points to a correlation between sustained elevated IOP and an increased incidence of ischemic optic neuropathy and visual loss (13).

An elevation in IOP during RALP as a risk factor for subsequent visual impairment was evaluated in two recent studies (14, 15). In a prospective study examining 19 patients with a history of glaucoma or ocular hypertension, no adverse ocular sequelae were reported postoperatively (14). In an additional study, patients were randomized into two groups receiving different anesthetic agents, with changes in IOP during the procedure as the primary measured outcome (16). Patients receiving balanced anesthesia with volatile anesthetics had significantly increased IOP compared to the group who received total intravenous anesthesia with propofol and remifentanil. The authors surmise that this could potentially have profound effects on patients with preexisting elevated IOP (14). In our patient groups we did not routinely record IOP during the pre, post or perioperative periods and, as such, it is unclear as to whether changes in IOP contributed to the incidence of retinal and CNS-related complications in our study.

Our data indicate that visual impairment and CNS-related complications following RALP surgery are relatively rare events. Nonetheless, in RALP patients, these post-surgical complications have the potential to significantly impact quality of life (17–19). The risk appears to be elevated in spine and cardiac surgery where anemia and hemodynamic changes increase the risk of ischemia (13). Due to the rarity of the event, however, the specific pathogenesis is still unclear.

A limitation of our study is the relatively small number of patients in our study group who were hypothesized as being at higher-risk of retinal and CNS-related complications following RALP due to preexisting relevant comorbidities. The relationship between the time spent in the Trendelenberg position and the incidence of retinal or CNS-related symptomatology is unclear. The absence of retinal or CNS-related complications could be due to the operative time being shorter than the time required to elicit retinal or CNS-related complications.

While the rates of other complications were also low, there was not a statistically significant difference in the incidence or severity of total complications between the control and study group. The relative experience of our surgical team with this robotic procedure may have had a significant (and positive) effect on limiting the incidence of perioperative complications and supports the notion of time in the Trendelenberg position and overall operative time as key indices in optimizing patient outcome. In our study, the single surgeon has more than 10 years experience of performing RALP, and operative times are relatively short and consistent. As such, data from our study may not necessarily be applicable for surgeons who have less experience in robotic surgery or are still learning and, as such, may have longer operative times.

The risks associated with sustained elevations of IOP on visual acuity may be particularly relevant for surgeons during their initial learning curve when surgical procedure times are prolonged. Furthermore, procedures such as robotic cystectomy may put patients at higher risk for retinal and/or CNS-related complications since the operative times are substantially longer in the Trendelenberg position and surgical blood loss typically greater.

## CONCLUSIONS

Perioperative ischemic optic neuropathy and CNS complications are a relatively rare event associated with RALP. Our study suggests that patients with a history of retinal and CNS pathology electing to undergo RALP are not at a significantly elevated risk of experiencing retinal and CNS-related complications. However, studies with larger patient cohorts are required to further support these data.

## ABBREVIATIONS

RALP: = Robot assisted laparoscopic radical prostatectomies
CNS: = Central nervous system
CVA: = Cerebrovascular accident
IOP: = Intraocular pressure
PSA: = Prostate specific antigen
CCI: = Charlson comorbidity index
BMI: = Body mass index
PCa: = Prostate cancer
IQR: = Interquartile range
IRB: = Institutional review board
CAD: = Coronary artery disease
CHF: = Chronic heart failure
MI: = Myocardial infarction
COPD: = Chronic obstructive pulmonary disorder
ICP: = Intracranial pressure
EBL: = Estimated blood loss
